# Simplified Calculation Model and Experimental Validation of the Force Transfer Ratio of Steel–Concrete Joint of Hybrid Box Girder

**DOI:** 10.3390/ma16145091

**Published:** 2023-07-19

**Authors:** Haibo Wang, Haozhe Zeng, Xun Wu, Fangcong Yu

**Affiliations:** School of Civil Engineering, Central South University, Changsha 410075, China; haozhezeng@outlook.com (H.Z.); fangcong_yu@163.com (F.Y.)

**Keywords:** force transfer, steel–concrete joint, simplified calculation model, the deformation coordination theory, model test

## Abstract

The axial force transfer ratio of steel–concrete joints in hybrid box girder bridges is crucial for bridge design. However, the current standard oversimplifies the transfer ratio distribution coefficients, and both model tests and finite element analysis are time- and labor-intensive. This article proposes a simplified calculation model based on the deformation coordination theory to estimate the transfer ratio of the axial force between the bearing plate and shear connectors of the steel–concrete joint under compression bending conditions. Additionally, a large-scale model (1/5 scale) is established, and the mechanical properties of the steel–concrete joint section under compression-bending conditions are experimentally tested. A three-dimensional finite element model is developed and verified using the obtained test data. Results confirm the favorable mechanical properties and ample safety reserve of the SCJ, with all components remaining within the elastic stage under 1.6 times design conditions. By comparing the axial force transfer ratios obtained from the simplified calculation model and the finite element model, a small difference is observed, validating the reliability of the simplified calculation model. This paper provides a straightforward and efficient method for the design and evaluation of steel–concrete joints in hybrid box girder bridges.

## 1. Introduction

A hybrid box girder cable-stayed bridge is a bridge structure that combines the use of both steel and concrete materials [1]. Steel box girders are utilized in the main span section to enhance the bridge span due to their lightweight nature, high strength, rapid construction, and ability to circumvent the drawbacks associated with concrete materials, such as self-weight, limited tensile capacity, and susceptibility to cracking [2,3,4]. On the other hand, PC (Prestressed Concrete) box girders are employed in the side span section to provide support, ballast, and numerous advantages such as high stiffness, stability, cost-effectiveness, fatigue, and corrosion resistance [5,6]. This application of PC box girders increases the overall stiffness of the bridge, reduces the amplitude of live load stress, mitigates the negative side span reaction force, and lowers costs [2,5,6].

Moreover, hybrid girder cable-stayed bridges offer excellent construction adaptability, enabling simultaneous construction of the cable towers and concrete box girders for the side spans. These bridges have gained widespread usage worldwide, featuring notable examples such as the Kurt Schumacher Bridge in Germany [7], the Normandy Bridge in France [8], the Russian Island Bridge in Russia [9], the Tatara Bridge in Japan [10], and the Yongjiang Bridge in China [5]. Table 1 presents the top ten main spans of hybrid girder cable-stayed bridges globally.

Hybrid girder box bridges are an innovative form of bridge structure with many advantages. However, the design difficulty of hybrid girder bridges lies in the connection area between steel and concrete girders, i.e., the steel–concrete joint (SCJ) [5,11,12,13]. At the SCJ, the sudden change in material and section stiffness on both sides leads to structural discontinuity and structural weakness, which may affect the safety of the bridge system [14,15,16]. Therefore, ensuring that the SCJ has good mechanical properties and making the stiffness and force transfer changes smooth is a key issue in the design of hybrid box girder bridges.

The structural configuration, mechanical properties, and durability of the steel–concrete joint (SCJ), which serves as the connection between the steel and concrete girder sections in the main beam, have received significant attention. Numerous studies have been conducted to examine the structural feasibility, stress distribution, force transfer behavior, slip characteristics, bearing capacity, stiffness variation, and fatigue damage of the SCJ. These studies provide theoretical foundations and technical guidance for SCJ design.

For instance, Chen Kaili et al. [17] conducted model tests to analyze the stress distribution and stress mechanism of the combined section. The tests verified that the SCJ of the Taoyoumen Highway Bridge remained elastic under 1.7 times the maximum axial force, shear force, and bending moment combinations. Huang Caiping [18] conducted experiments to study the load characteristics and bearing capacity of shear connectors in the composite section. They proposed the “rubber-shear stud” combined connector and derived formulas for its bearing capacity. The research showed that the diameter of the shear stud has the greatest impact on its load-carrying capacity. It directly determines the shear resistance and stiffness of the shear stud. Reference [13] conducted model tests and finite element analysis on the SCJ section of Wusu Bridge on Hei Xiangzi Island, confirming the smooth transfer of loads through the SCJ and ensuring that the stresses in the SCJ members met the material strength requirements. In reference [15], model tests were performed on the SCJ of the Nujiang Second Bridge in Liuku, Yunnan, discussing the structural response of the large-scale model under combined axial force, bending moment, and torque. The test results demonstrated that the SCJ containing UHPC (Ultra-High-Performance Concrete) exhibited favorable mechanical properties and sufficient strength, with the stresses in each member primarily determined by the axial force and bending moment, while the influence of torque was not significant. In reference [16], a 1:2 scale model test was conducted on an actual hybrid girder cable-stayed bridge project. The test results revealed that under 1.5~1.7 times ultimate load combination conditions, the specimen remained in the elastic stage without evident damage such as concrete cracking, steel plate buckling, or interface slippage. The section displayed excellent structural performance and had a certain safety margin.

Regarding the construction and force performance of the SCJ in the main girder of a large-span cable-stayed bridge for railways, references [5,14] focused on Yongjiang Railway Bridge, discussing reasonable construction techniques and fatigue damage of the SCJ through model tests and finite element simulation analysis. Yang et al. [19] investigated the force and deformation behavior of the SCJ in the Tanjiang Special Bridge of the Shenmao Railway, verifying its satisfactory performance. Xin H., Liu Y., et al. [20] conducted model tests on five specimens with different types of stiffening ribs in the stiffening transition section of the SCJ. They studied the ultimate bearing capacity and damage characteristics of these specimens, finding that embedded T-type stiffening ribs exhibited superior axial stiffness, while external π-type stiffening ribs showed excellent out-of-plane stiffness and ultimate bearing capacity. Reference [11] conducted tests on five SCJ lattice chamber specimens, modifying parameters such as the bearing plate thickness, shear connector distribution, and contact relationship between the bearing plate and filled concrete. By loading the specimens until complete destruction, the model tests analyzed the internal force transfer mechanism and revealed the load distribution ratio between different components of the bonded section.

In summary, the existing research on SCJs demonstrates their significant safety reserve capacity for both highway and railroad bridges, making them suitable as critical connection components between the steel box girder section and the PC girder section in hybrid girder bridges. However, the current research methods for SCJs primarily involve reduced-scale model tests that retain the cross-sectional characteristics of the original structure, coupled with finite element analysis. While these methods have provided valuable insights into the macro structural performance of the combined section and the axial force transfer ratio of each component, they also have limitations. Model tests are time-consuming, and finite element analysis requires substantial computational resources. Furthermore, the current method for estimating the maximum shear force in the SCJ, as outlined in the Specifications for Design and Construction of Highway Steel-concrete Composite Bridges [21], is relatively crude. It solely relies on a force distribution coefficient to determine the axial force transfer ratio of the bearing plate, without considering the influence of different bridge configurations. This approach deviates significantly from the results obtained through model tests and finite element analysis. Given the crucial role of the axial force transfer ratio in SCJ design, it is necessary to propose a simplified calculation model for accurately determining this parameter.

The use of model tests, finite elements, and numerical investigation is very widely used in the principal research of civil and structural engineering [22,23]. Therefore, this article presents a simplified calculation model for the force transfer ratio of the SCJ, which is based on the deformation coordination theory. The aim is to address the limitations of the current code’s method for calculating the axial force transfer ratio in the SCJ. To verify the reliability of the proposed simplified model, 1:5 scaled-down model tests and finite element analyses are conducted, using the cross-Yujiang railway bridge as the engineering background.

## 2. Experimental Program

### 2.1. Test Model Design

**Figure 1:**
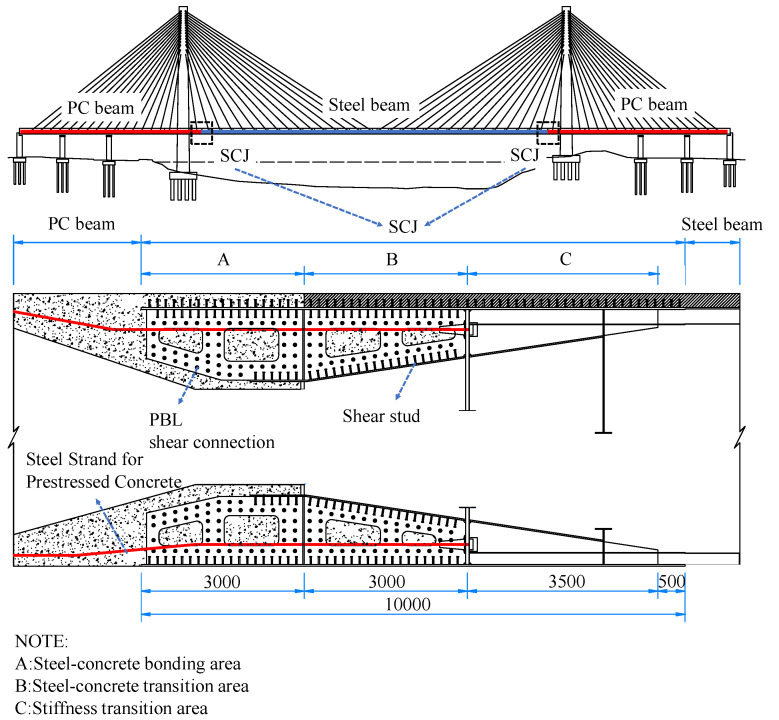
Location of the SCJ and structure of the SCJ of the actual bridge (unit: mm).

The purpose of this experiment is to investigate the mechanical properties of the steel–concrete joint (SCJ) in the cross-Yujiang railway bridge. Figure 1 illustrates the precise location of the SCJ in the actual bridge, along with its structural configuration and the adjacent steel box girders and PC box girders. To accurately simulate the force conditions experienced by the SCJ, a scaled-down test model was meticulously designed using scaling theory and St. Venant’s principle. When selecting the scale for the model tests, three scales were considered: 1:4, 1:5, and 1:6. However, due to limitations in the size of the test site and the capacity of the loading equipment, conducting tests at a scale of 1:4 would be challenging. On the other hand, using a scale of 1:6 would require obtaining an extremely thin steel plate for model construction, which proved to be difficult. Moreover, such a thin steel plate could potentially result in significant distortions in the test model. As a result, a scale of 1:5 was deemed to be the most feasible option for conducting the tests.

In order to eliminate the effect of size effect, four similarity criteria are considered when scaling according to similarity criteria. (i) Steel plates: apply stress equivalence criterion for downsizing. (ii) Steel reinforcing bars: retain reinforcement ratio. (iii) Post-tensioned tendons: apply stress equivalence criterion for equivalent prestressing effects. (iv) Shear connectors: apply area equivalence criterion for equivalent shear behavior. Table 2 provides a comprehensive comparison between the test model and the physical properties of the actual bridge, as derived from the principles of similarity theory conversion.

**Figure 2:**
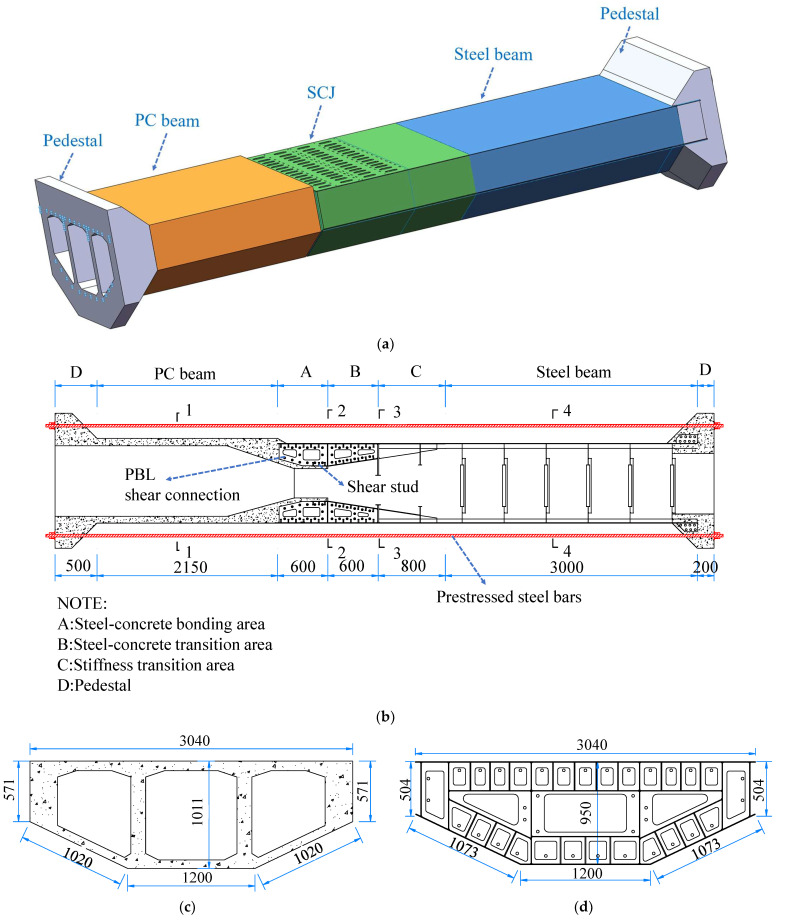

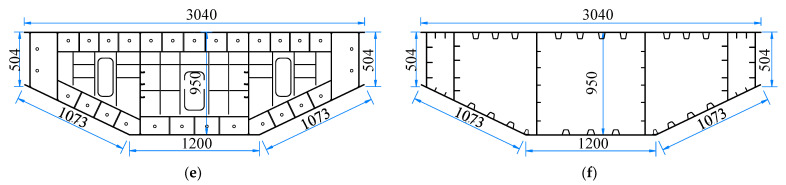
Test model (unit: mm): (**a**) model structure; (**b**) general arrangement of model box girder center; (**c**) concrete box girder section (1-1 section); (**d**) front bearing plate section (2-2 section); (**e**) rear bearing plate section (3-3 section); (**f**) steel box girder section (4-4 section).

To account for the influence of St. Venant’s principle, the test was designed to scale down not only the SCJ structure but also some adjacent steel box girders and PC box girders. This also makes the boundary conditions of the SCJ more realistic and further eliminates the effect of size effect. During the downsizing process, the complexity of the original bridge’s windjammer posed challenges in constructing an accurate scaled-down model. Consequently, the air nozzles, which had minimal impact on the structural performance, were removed [24]. Furthermore, since the test employed the self-anchored loading method (refer to Section 2.2 for more details), pedestals were added at the ends of the steel box girder and PC box girder in the test model to prevent the loading end load from affecting the stresses near the study area. Figure 2 provides a visual representation of the test model.

During this test, efforts were made to maintain consistency in the specification types of the main plates and shear connectors between the actual bridge and the scaled-down test model at a ratio of 1:5. Table 3 provides a detailed comparison between the two. However, due to certain material specifications not being readily available in the market, slight adjustments were made to the plate thickness of some components in the test model after the scaling-down process. Additionally, detailed construction modifications were implemented to ensure that the section characteristics of the control section (as described in Section 2.2 of this article) were maintained. These adjustments were made to ensure that the overall force distribution and force transmission path of the test model remained consistent with the actual bridge.

For this test, efforts were made to ensure that the materials used in the test model’s concrete and steel structures closely matched the actual bridge. Table 4 provides a comparison of the materials used in both cases. However, due to challenges in procuring bridge steel q345qD in the market and specific thickness requirements for the model steel plate in this test, Q355B grade steel was used as a substitute. The mechanical properties of Q355B grade steel are comparable to Q345qD grade steel. It is important to note that this test is conducted in a laboratory setting where the ambient temperature remains above 0 °C. Therefore, there will be no impact on the low-temperature toughness of the steel plate during the test. As a result, the use of Q355B grade steel instead of Q345qD grade steel is feasible under these circumstances.

**Figure 3:**
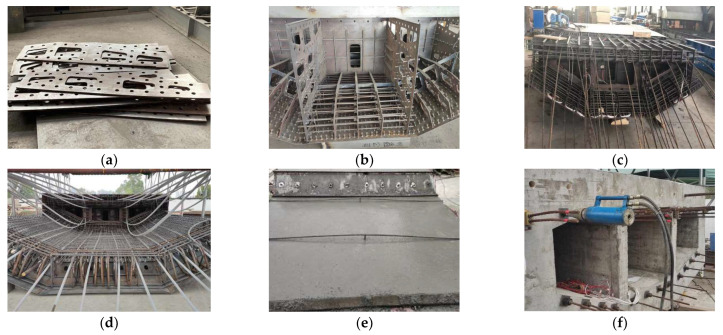
Test model processing process: (**a**) plate processing; (**b**) steel structure assembly; (**c**) preset prestressing tendons; (**d**) tying reinforcement; (**e**) concrete pouring; (**f**) tensioning prestressing strands.

The test model used in this study consists of both steel and concrete components. Figure 3 illustrates the fabrication process of the test model. The steel structure was manufactured in a qualified steel plant following the provided drawings, and a quality inspection report was issued to ensure its compliance. The concrete part, on the other hand, was processed at the laboratory testing site where the structural tests were conducted. Table 5 shows the test results of the test model steel material properties.

Steel formwork was employed for the bottom surface of the test model and the outer side of the side webs. Stiffening ribs were welded onto the steel formwork to maintain its stiffness and prevent deformation. Wooden formwork was utilized within the test model and at the loading end to facilitate formwork adjustment. Once the reinforcement cage was assembled, concrete was poured into the formwork.

Considering that the casting took place during winter with a laboratory temperature below 5 °C, the test model was covered with plastic film for proper curing after casting. After 5 days, the formwork was removed, and prestressing was applied after 28 days. Compressive strength tests were conducted on concrete cubes taken from the same concrete batch and cured under identical conditions. These tests ensured that the concrete had attained sufficient strength prior to prestressing. Table 6 shows the test results of the test model concrete material properties.

### 2.2. Loading Program

**Figure 4:**
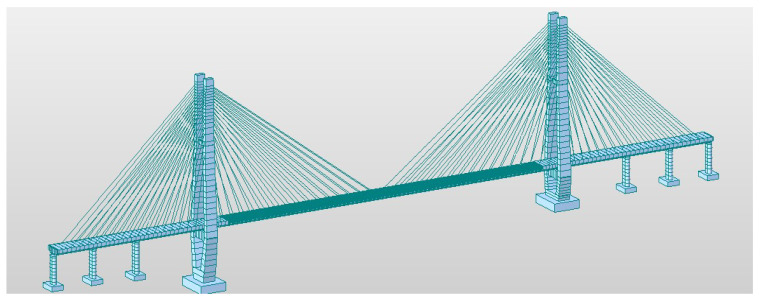
MIDAS model of the actual bridge.

Figure 4 depicts the MIDAS finite element model of the actual bridge, which is discretized into 849 units and 1026 nodes. The model’s load arrangement follows the guidelines outlined in the General Code for Design of Highway Bridges and Culverts [25]. This arrangement encompasses various load types, including main forces (such as constant and live loads), additional forces (such as braking or traction forces, wind loads, temperature effects, and flowing water pressure), and special loads (such as seismic forces and flowing water pressure).

To facilitate the selection of test loads, the control section for internal forces is chosen at the interface between the standard steel box girder and the stiffness transition zone. The finite element model provides the internal forces for the control section under different load cases, including constant load, live load, and longitudinal wind. These internal forces are presented in Table 7, where the axial force is considered positive in tension, the bending moment is considered positive in the lower tension zone, and the shear force is considered positive in a clockwise rotation around the isolated body.

Based on the data provided in Table 7, it can be observed that the shear force and torque applied to the SCJ are significantly smaller compared to the bending moment and axial force. As a result, when selecting the unfavorable load case for testing, the axial force and bending moment are primarily considered as the controlling conditions, while the effects of shear force and torque are neglected. Three loading conditions were considered for the test model based on the data in Table 7: maximum axial force, maximum negative bending moment, and maximum positive bending moment. These loading conditions are chosen to represent critical scenarios and evaluate the performance of the SCJ under different loading conditions.

**Figure 5:**
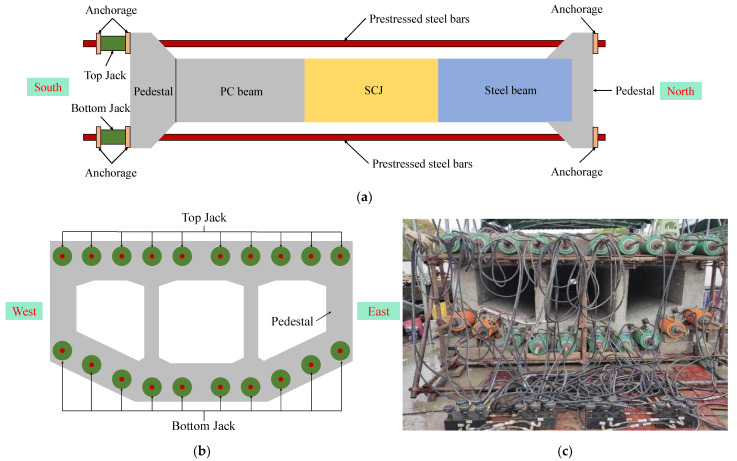
Loading method of the test model: (**a**) elevation view; (**b**) left view; (**c**) photograph of the loading equipment.

Figure 5 illustrates the self-anchored loading method employed for the test model. Pedestals were constructed at the ends of the steel box beam girders and PC box girders, and they were poured simultaneously with the PC box girders. Ten prestressing steel bars were threaded into each side of the pedestals, with one end anchored in the pedestal on the steel box girder side. The other end of the bars passed through the pedestal on the PC box girder side and extended into the through-core jack. The prestressing steel bars were also anchored at the other end of the jack.

During the loading process, tension is applied to the prestressing steel bars using the jack. As a result, the test model experiences compression due to the anchoring of the prestressing steel bars at both ends, enabling axial force loading. By adjusting the pressure difference between the jacks on the top and bottom sides of the base, the imbalance of the axial forces on the top and bottom sides of the test model can be controlled, facilitating the application of bending moment loading. Table 8 shows the total force applied by the jack under different load cases.

The test was conducted using a graded loading method, following the load case presented in Table 9. The load value corresponding to 1.0 times the working condition was denoted as P. Prior to the test, the specimens underwent two preloading stages at 0.2 P and 0.4 P. During the test, the specimens were subjected to loading conditions at 1.0 and 1.6 times the maximum axial force, maximum positive bending moment, and maximum negative bending moment. Each loading stage accounted for 20% of the load at 1.0 times the working condition. The loading sequence involved applying pressure to the top loading end first, followed by the bottom loading end. Once each loading level was completed and the strain data reached stability, the strain and displacement at the test measurement points were recorded. Additionally, the slip between the steel structure and the concrete was measured.

### 2.3. Measurement Programs

**Figure 6:**
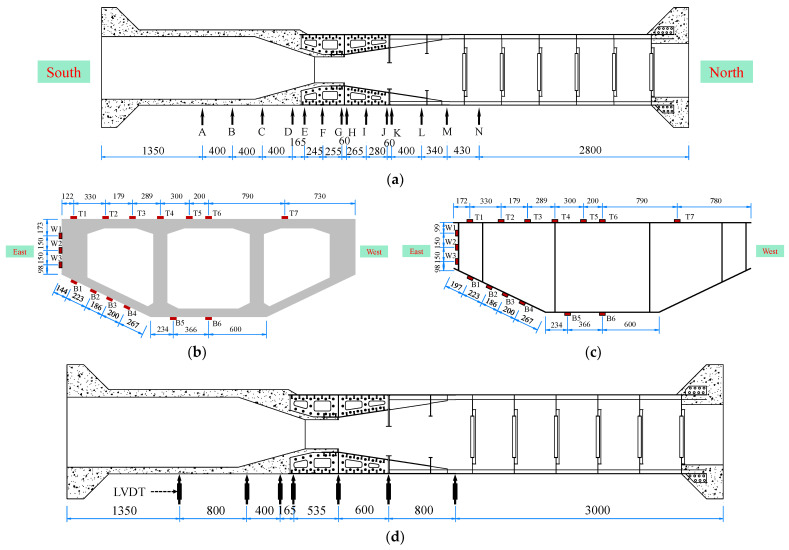
Measurement equipment layout (unit: mm): (**a**) strain gauge layout section; (**b**) A–D section strain gauge layout; (**c**) E–N section strain gauge layout; (**d**) LVDT layout location.

Figure 6 illustrates the arrangement of measurement instruments for the test model. Resistive strain gauges were utilized for strain measurement, and data acquisition was performed using strain acquisition systems. The strain gauges were strategically positioned in 14 cross-sections, encompassing the steel box girders, SCJs, and PC box girders. The strain gauges were labeled according to “section position + deployment position in the section + position of the strain gauges in the section”. For example, “AT1” indicates the strain gauge located at the top plate of section A, position 1.

To measure displacement, seven linear variable differential transformers (LVDTs). These LVDTs were installed along the centerline of the box girder at the model’s bottom and covered the steel, SCJ, and PC box girders. Additionally, a micrometer was utilized to measure slips at the steel mixed junction. The micrometer was affixed to the steel structure of the SCJ, with its measuring end in contact with the concrete surface. The slip value was obtained by monitoring changes in the micrometer reading.

## 3. Results

### 3.1. Stress Distribution in the Longitudinal Direction

To accurately describe the stress distribution of the test model in the 1.0 Nmax, 1.0 Mmax, and 1.0 Mmin cases, the following guidelines are established for data analysis:

1. Tensile stress is considered positive, while compressive stress is considered negative;
2. The analysis is based on the position of the front bearing plate of the SCJ as the coordinate origin. Positive values indicate the direction toward the steel box beam side, while negative values indicate the direction toward the PC box beam side;
3. A distance of −0.6 m from the front bearing plate corresponds to the intersection between the PC box beam and the SCJ;
4. The position of the rear bearing plate is located at a distance of 0.6 m from the front bearing plate;
5. A distance of 1.4 m from the front bearing plate corresponds to the intersection between the SCJ and the steel box beam.

By adhering to these provisions, the stress distribution within the test model can be clearly and intuitively described, enabling a comprehensive analysis of stress levels and transmission mechanisms in the 1.0 Nmax, 1.0 Mmax, and 1.0 Mmin cases.

**Figure 7:**
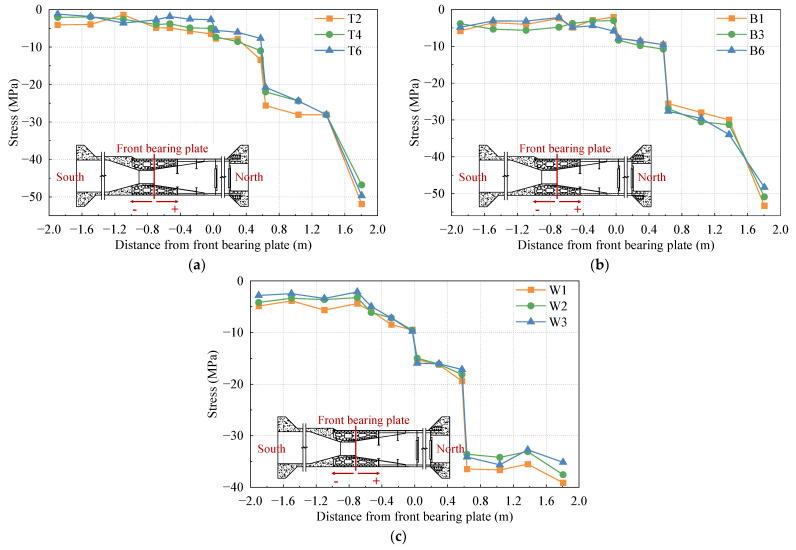
Stress distribution under the 1.0 Nmax case: (**a**) top plate; (**b**) bottom plate; (**c**) side webs.

**Figure 8:**
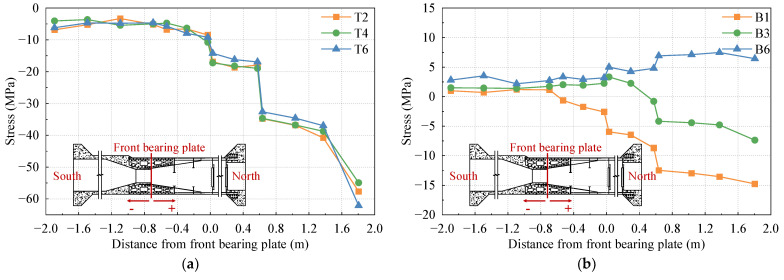

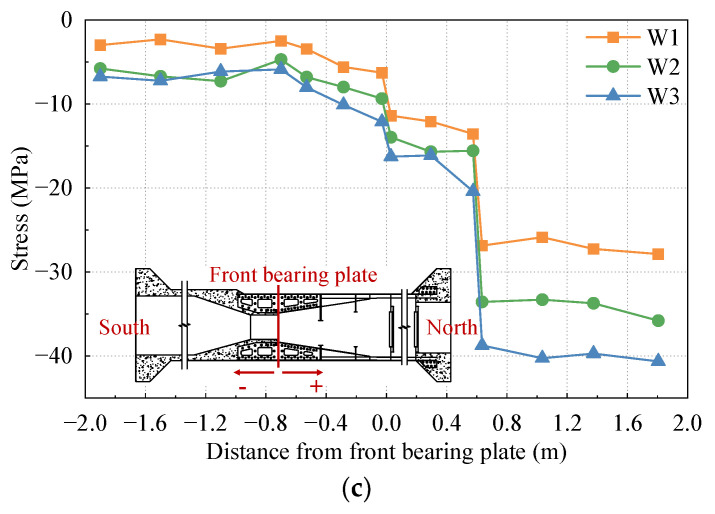
Stress distribution under the 1.0 Mmax case: (**a**) top plate; (**b**) bottom plate; (**c**) side webs.

**Figure 9:**
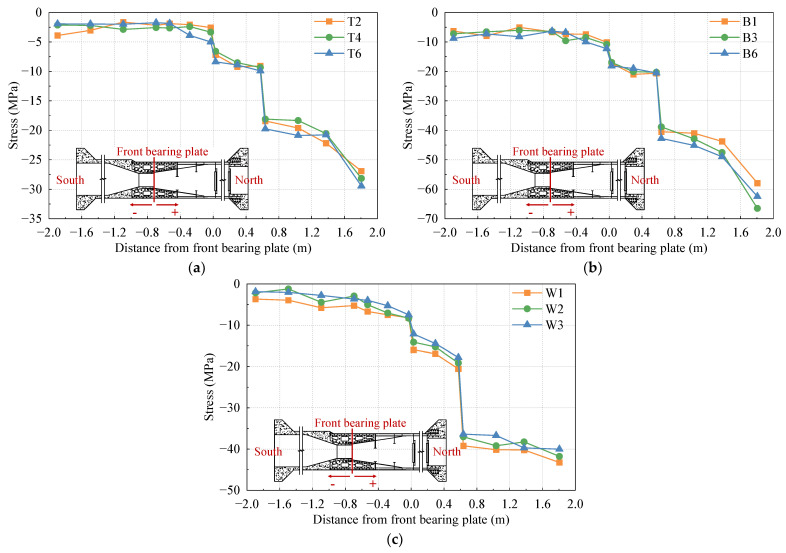
Stress distribution under the 1.0 Mmin case: (**a**) top plate; (**b**) bottom plate; (**c**) side webs.

To analyze the stress distribution of the top plate, bottom plate, and side web of the test model under different cases, several columns of strain gauges are arranged along the longitudinal direction of the bridge. The representative data from these strain gauges are selected for detailed discussion. The stress distribution curves of the top plate, bottom plate, and side web under the 1.0 Nmax, 1.0 Mmax, and 1.0 Mmin cases are shown in Figure 7, Figure 8, and Figure 9, respectively.

In Figure 8, it is evident that under the maximum positive bending moment case, the steel–concrete transition areas and steel–concrete bonding areas are primarily under tension. However, the bottom plate in the stiffness transition area experiences partial compression. The overall change in stress value is relatively small, which can be attributed to the fact that the load applied by the top jack is significantly greater than the load applied by the bottom jack, leading to tension in part of the bottom plate.

In Figure 7, Figure 8 and Figure 9, it is evident that the compressive stress values of the top slab, bottom slab, and side webs exhibit significant variation at both sides of the front and rear bearing plates. The rear bearing plate, in particular, shows a substantial effect in transferring most of the internal forces to the concrete of the SCJ. In the PC box girder section (located on the left side at −0.6 m from the front bearing plate) and the steel box girder section (located on the right side at 1.8 m from the front bearing plate), the stress levels in each plate are lower and more uniformly distributed. Due to the difference in thickness between the top plate of the steel box girder section and the SCJ, significant changes in stress levels are observed before and after the steel box girder section line, while the side webs exhibit less pronounced stress changes due to their consistent thickness.

### 3.2. Vertical Displacement

**Figure 10:**
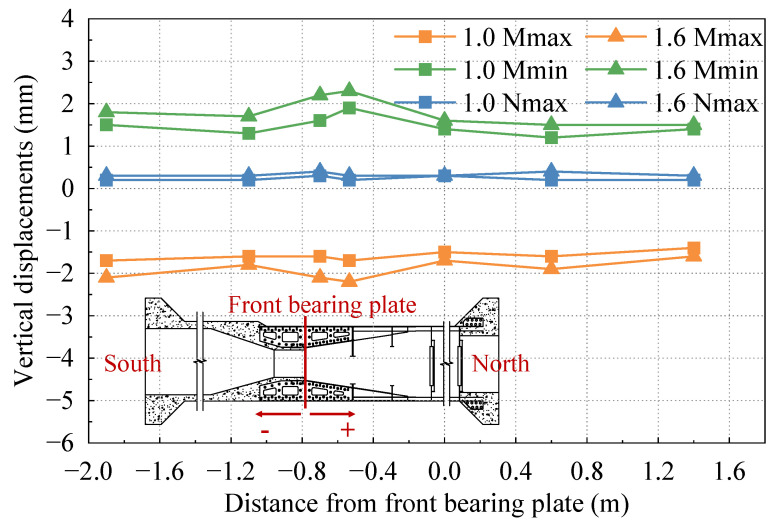
Vertical displacement under different cases.

Figure 10 illustrates the vertical displacements of the test model under different cases. Positive values in the picture indicate downward vertical displacement and negative values indicate upward vertical displacement. It can be observed that the vertical displacement ranges from −2.2 mm to 2.3 mm, exhibiting a relatively consistent change trend. Within each case, the vertical displacements at various positions do not differ significantly, and there are no abrupt changes in displacement. Slightly larger displacement variations are observed at the intersection of the PC box beam and SCJ, which can be attributed to the differing stiffness at this junction. Nevertheless, the vertical displacements at this intersection and other positions remain relatively close, and the secondary moment effects on the structure are negligible. Overall, the vertical deformation of the test model appears continuous and smooth, with a smooth transition in stiffness. 

### 3.3. Relative Slip of Steel and Concrete

**Figure 11:**
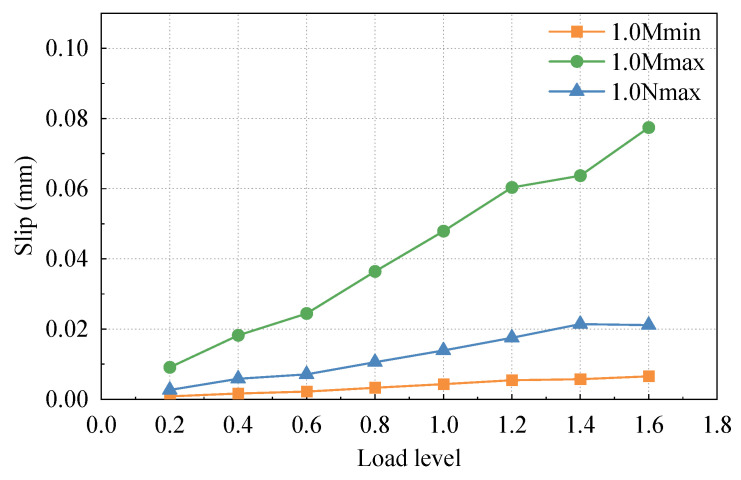
Load slip curve.

Figure 11 depicts the relative slip curves of steel and concrete under different cases with load. It can be observed that the relative slip between steel and concrete is greater under the maximum positive moment case compared to the other two cases. This is primarily due to the load applied at the upper loading end, where the upper jack applies the largest load during the maximum positive moment case. The relative slip between steel and concrete demonstrates a linear increasing trend as the load level increases. The load-relative slip curve appears relatively smooth, indicating that the slip between steel and concrete near the joint section remains in a linear state throughout the load range up to 1.6 times for various cases.

In general, the relative slip between steel and concrete near SCJ is small, the maximum value does not exceed 0.08 mm (1.6 times the maximum positive moment case), and the load relative slip curve is also relatively smooth, indicating that this test model has continuous and smooth deformation of steel and concrete and good working synergy.

### 3.4. Model Carrying Capacity Analysis

**Figure 12:**
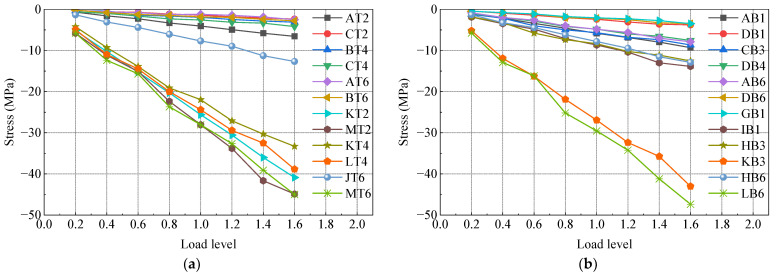

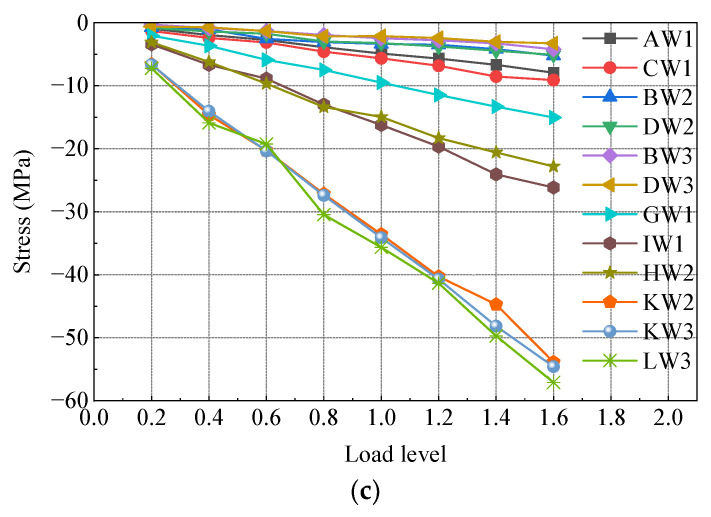
Load stress curve under maximum axial force case: (**a**) top plate; (**b**) bottom plate; (**c**) side webs.

**Figure 13:**
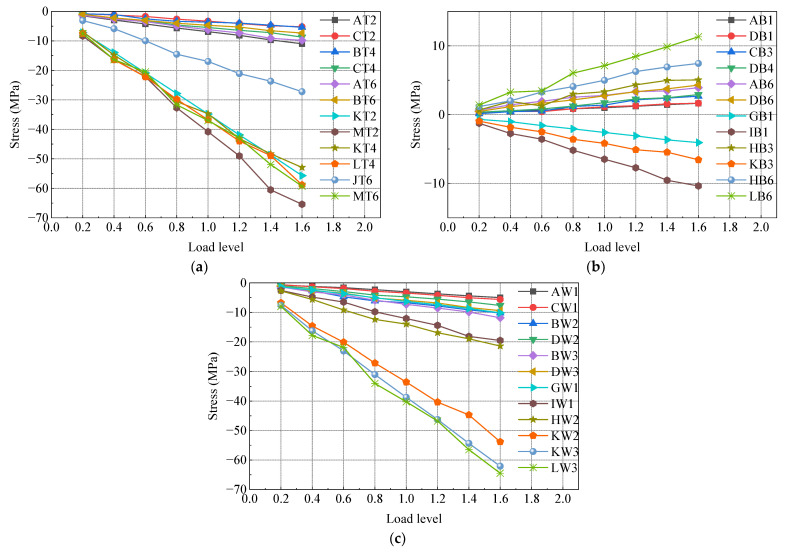
Load stress curve under maximum positive bending moment case: (**a**) top plate; (**b**) bottom plate; (**c**) side webs.

**Figure 14:**
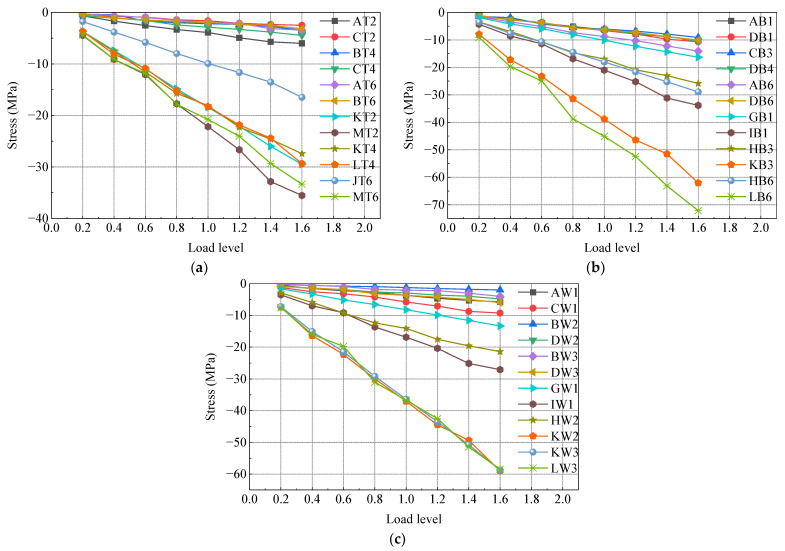
Load stress curve under maximum negative bending moment case: (**a**) top plate; (**b**) bottom plate; (**c**) side webs.

The test model underwent gradual loading up to 1.6 times the maximum axial force case, 1.6 times the maximum positive bending moment case, and 1.6 times the maximum negative bending moment case using a graded loading method. Figure 12, Figure 13 and Figure 14 depict the load stress curves of the top plate, bottom plate, and web plate with representative measurements under each case. The strain curves of each loading condition are grouped according to the strain gauge number. Due to the large number of measurement points, only part of the data is shown in the figure. In order to make the data representative, the selected strain gauges are distributed in various locations of the test model as much as possible.

In Figure 12, Figure 13 and Figure 14, it is evident that the stresses in the steel box girder section, SCJ, and concrete box girder section exhibit a linear relationship with the change in load under 1.6 times the maximum axial force case, 1.6 times the maximum positive bending moment case, and 1.6 times the maximum negative bending moment case. Furthermore, the stress levels in each member remain below the allowable stress of the material, indicating that each member is still in an elastic working state. Additionally, no buckling of the steel structure, significant deformation, or concrete cracking occurred during the test. These observations indicate that the SCJ of the test model is safe, reliable, and possesses sufficient safety margin.

## 4. Finite Element Analysis

### 4.1. Finite Element Model

**Figure 15:**
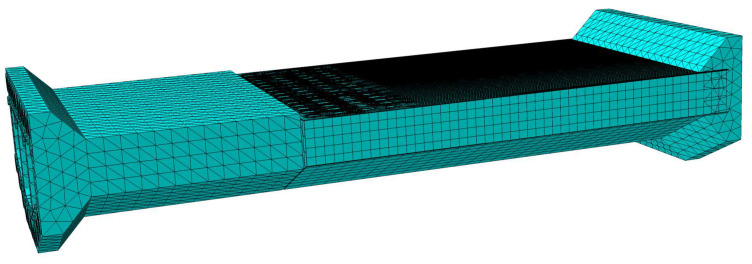
A 3D finite element model of the test model.

**Figure 16:**
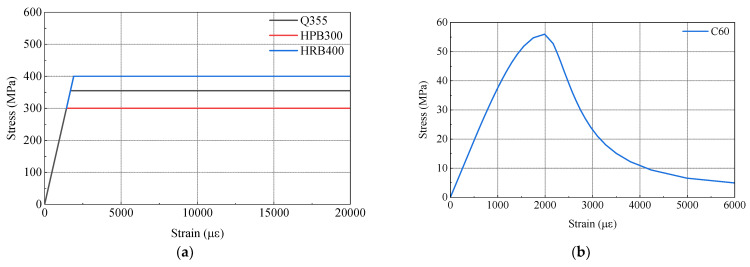
Constitutive models used for materials: (**a**) constitutive model for steel; (**b**) constitutive model for concrete.

To gain a more comprehensive understanding of the stress distribution in the test model and investigate its mechanical properties, a finite element model was developed using ABAQUS 2020 software, as depicted in Figure 15. Figure 16 illustrates the constitutive models employed in the finite element model. The material properties in the model are consistent with those used in the physical test, ensuring the reliability of the model. For the steel components, an ideal elastoplastic principal model was adopted (as shown in Figure 16a), wherein the strain exhibits a linear relationship with increasing stress until reaching the yield point, beyond which the stress value remains constant. The plastic damage intrinsic model [26] was employed for the concrete material (as shown in Figure 16b).

The finite element model incorporates different element types to accurately simulate the components of the test model. The steel plate is represented by CSS8 continuous solid shell units, while the concrete is simulated using C3D8R and C3D10 solid units. The PBL reinforcement and shear studs are modeled using C3D8R solid units, and the prestressing strands and various steel bars are represented by T3D2 truss units.

To capture the welding between the steel plates, a tied constraint is applied. The bond–slip relationship between the concrete and the top plate, bottom plate, side web, and rear bearing plate in the steel mixed combined section is simulated using a surface-to-surface contact relationship. The normal contact relationship is set to hard contact, and the tangential contact relationship is governed by a penalty function with a friction coefficient of 0.5 [27].

To simulate the joint action between the steel plates, shear studs, common reinforcement, prestressing strands, and PBL reinforcement, which are embedded within the concrete, an embedded built-in area constraint is employed. The bottom surface of the shear stud is constrained with tied constraints to the steel plate surface. The PBL reinforcement is split at the openings of the PBL plate ribs, and the corresponding surfaces of the PBL plate ribs are constrained with tied constraints to the surface of the corresponding PBL reinforcement. Location tolerances are specified to simulate the joint action between the PBL reinforcement and the PBL plate ribs [28].

The finite element model consists of a total of 236,880 elements and 472,331 nodes, capturing the complex interactions and behaviors of the test model.

To simulate the anchorage of the prestressing strand and prevent stress concentration, a rigid mat is placed at the anchorage end of the prestressing strand. Ties are used to bind the concrete beam, mimicking the anchorage of the prestressing strand and ensuring stress distribution. The interaction between the prestressing strand and the anchorage is simulated by binding the prestressing strand to a reference point located above the rigid mat using tie restraints. The prestressing stress within the prestressing strand is applied using the cooling method [29].

The boundary conditions of the finite element model are based on the actual boundary conditions in the model test. The displacement/cornering method is employed to restrict displacements in the X, Y, and Z directions at the bottom of the concrete box girder section of the model’s transmission structure. Similarly, displacements in the X and Z directions at the bottom of the steel box girder’s transmission structure are constrained, thereby simulating the simply supported beam configuration of the test model.

The finite element model consists of four analysis steps. In the initial analysis step, the temperature field is applied to the prestressing strand, and the static general module is used to apply gravity in the Z direction to the test model. In the second analysis step, the temperature field value of the prestressing strand is adjusted to induce prestress within the strand using the temperature field difference. In the third analysis step, compression force is applied to the steel spacers at both ends of the model, simulating the load applied by the jack. This is achieved by applying pressure to the steel mat at both ends of the model. The corresponding load values match those applied in the test model.

### 4.2. Finite Element Model Validation

**Figure 17:**
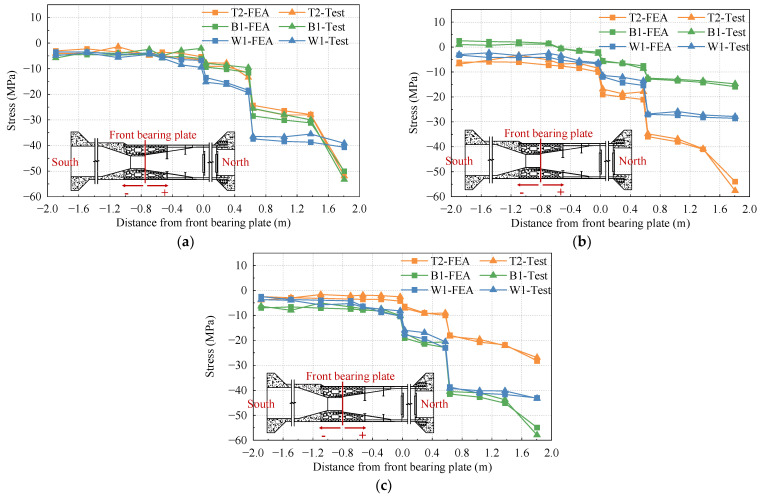
Comparison of finite element calculation value and test value: (**a**) 1.0 Nmax case; (**b**) 1.0 Mmax case; (**c**) 1.0 Mmin case.

To assess the accuracy of the finite element analysis, a set of measurement positions was selected on key components of the test model. The measured stress values obtained from the physical test were then compared with the stress values calculated by the finite element model, without considering the prestress effect. This comparison aimed to determine the agreement and trend of stress levels between the measured and calculated values. The results of this comparison under each working condition are presented in Figure 17.

Based on Figure 17, it is evident that the stress levels and variations of each member in the SCJ under different working conditions show good agreement between the measured data from the test and the calculated values from the finite element model. However, certain measured points exhibit some errors, which can be attributed to several factors. These include potential welding defects and concrete casting imperfections during the manual assembly and welding of the test model, possible errors in the loading and testing equipment, and the fact that the idealized finite element model may differ from the actual conditions. Nevertheless, the consistency between the measured data and the calculated values confirms the reliability of the finite element model.

### 4.3. Analysis of the Axial Force Transfer Mechanism of Concrete and Steel Structures

**Figure 18:**
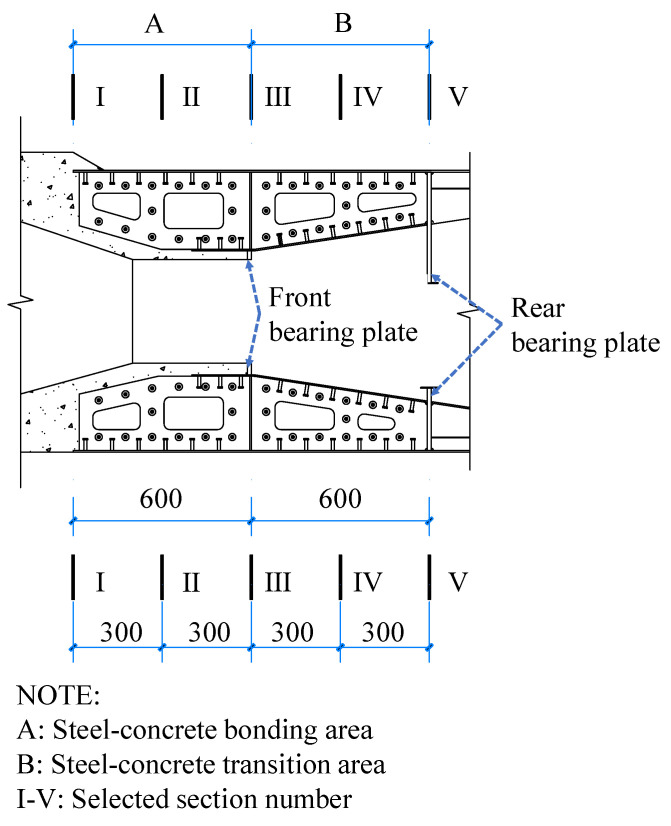
Cross-section selected for axial force transfer ratio analysis.

**Figure 19:**
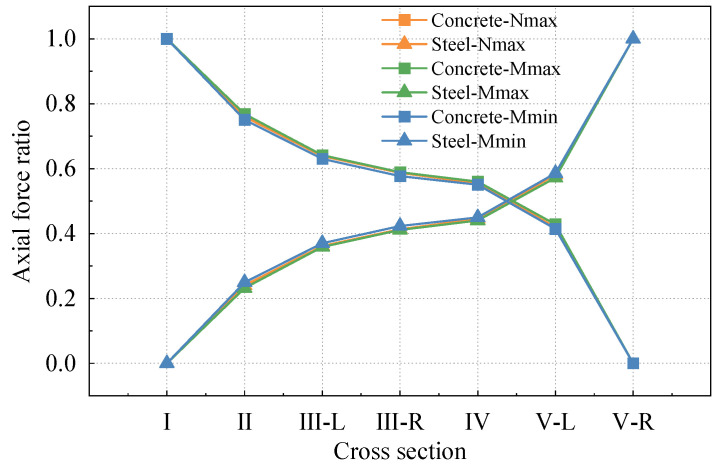
Axial force transfer ratio between steel and concrete structures.

To investigate the axial force distribution in the steel and concrete structures of the SCJ along the longitudinal direction of the bridge, three different working conditions were considered. A modified model, accounting for the prestressing effect, was utilized to analyze the longitudinal stresses of various members within specific sections (refer to Figure 18). The axial force transfer ratios between steel and concrete at these sections were determined through stress integration. The obtained results are presented in Table 10 and illustrated in Figure 19.

Figure 19 illustrates that the influence of different cases on the proportion of axial force transfer is minimal. This can be attributed to the SCJ being subjected to a complex force state within the bridge structure. Consequently, the SCJ design ensures symmetrical distribution of axial force transmission members at the top and bottom, minimizing the impact of the bending moment on the proportion of axial force transfer under different working conditions. 

Upon transferring the axial force from the stiffness transition zone to section V (rear bearing plate), the proportion of axial force carried by the steel structure decreases to approximately 58%, while the proportion borne by the concrete increases to around 42%. Hence, the rear bearing plate serves as the primary force transfer member, transmitting a significant portion of the axial force from the steel structure to the concrete within the steel mixed combination zone of the lattice chamber. This observation highlights the critical role of the rear bearing plate. 

Moving from section V to section I, the proportion of axial force borne by concrete gradually increases, while the proportion borne by the steel structure decreases. In section III (front bearing plate), the proportion of axial force borne by the steel structure decreases from approximately 42% to 35%, whereas the proportion borne by concrete increases from around 58% to 65%. This indicates that the front bearing plate also contributes to the transmission of axial force, albeit with a less pronounced effect compared to the rear bearing plate. This finding aligns with the conclusion drawn in reference [5]. 

The proportion of force transfer in other sections exhibits a smooth transition, suggesting a consistent force transfer mechanism within the steel–hybrid combination zone and steel–hybrid transition zone.

## 5. Simplified Calculation Model of the SCJ Axial Force Transfer Ratio

### 5.1. Calculation Method and Basic Assumptions

Based on previous theoretical and experimental studies [30,31,32], the load transfer in the SCJ is primarily accomplished through the relative slip effect between the steel and concrete in the shear studs and PBL shear connectors. To accurately calculate the transfer ratio of the shear keys in the bonded section, a simplified theoretical model is established, taking into account the pressure-bearing transfer effect of the bearing plate and the relative slip effect between the steel and concrete. This model is based on the load slip deformation coordination theory used in deriving the transfer mechanism of the PBL shear connector key group in reference [33].

The analysis results of this study indicate that the influence of bending moment on the proportion of axial force transfer is negligible. Furthermore, under pressure-bending conditions, the axial force transfer effect of the front bearing plate is not significant, as it remains in the elastic stage during normal use. To facilitate the establishment of simplified calculation equations, the following reasonable assumptions are made:

1. Both steel and concrete are assumed to be isotropic elastomers within the elastic force range;
2. The steel and concrete structures along the longitudinal bridge direction conform to the assumption of flat sections;
3. The shear joint is represented by an equivalent spring, where the shear force is proportional to the relative slip between the steel and concrete, disregarding the nonlinearity of the shear joint;
4. Adhesive friction between the steel and concrete is neglected;
5. Structural bending and shear deformation are disregarded;
6. The force transfer effect of the front bearing plate is ignored.

### 5.2. Theoretical Model of Deformation Coordination

**Figure 20:**
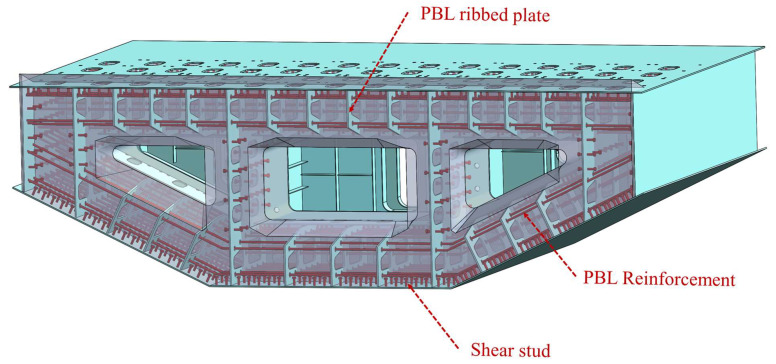
The SCJ internal structure.

**Figure 21:**
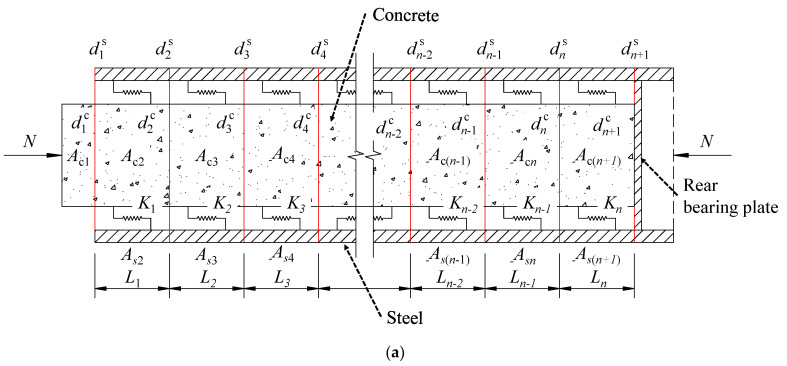

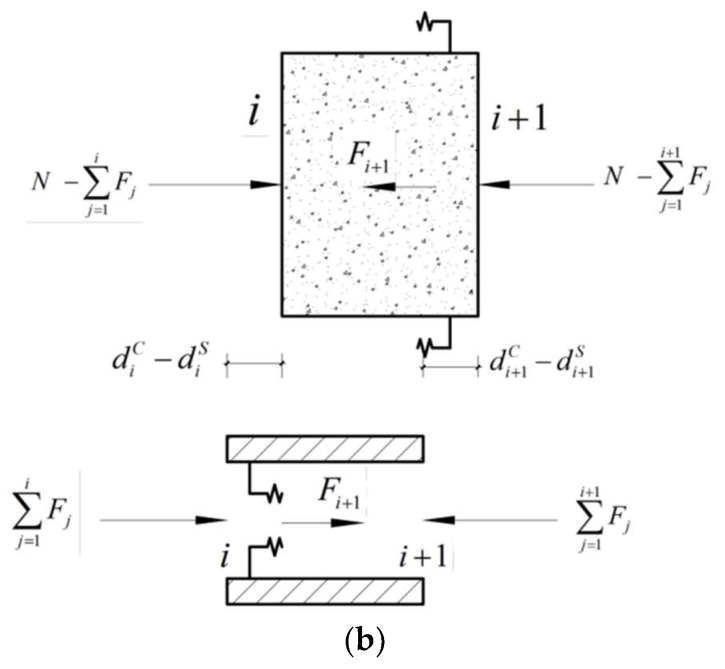
Simplified analysis of the SCJ: (**a**) simplified diagram of the SCJ as a whole; (**b**) simplified diagram of the SCJ segments.

The SCJ structure is shown in Figure 20. The transfer of axial forces in the SCJ is mainly accomplished through the local compression of the concrete by the bearing plate and the longitudinal shear action of the shear key group, so the force calculation of the SCJ can be simplified as shown in Figure 21. The compartment is divided into several sections. For section *i*, its length is *L_i_*, the section area of the steel member is *A*_s*i*_, and the section area of the concrete is *A*_c*i*_; the shear stiffness of segment *i* is *K_i_*, including the stiffness of the shear stud *K_di_* and the stiffness the PBL shear connection *K_pi_*; the absolute displacement of the end of the steel member of segment *i* is *d_i_^s^* and the absolute displacement of the concrete end is *d_i_^c^*; *F_i_* is the shared axial force of segment *i* and *N* is the total axial force transmitted by the SCJ; and *E*_s_ and *E*_c_ are the moduli of elasticity of steel and concrete, respectively.

Both the steel and concrete parts of the sections within the SCJ satisfy Hooke’s law, i.e., they satisfy Equation (1):

(1) $$\Delta l=\frac{F_{N}l}{EA}$$

Equation (1) and the equilibrium conditions of forces are utilized to derive the deformation coordination equations for the steel and concrete parts of section *i*. The equations are represented as Equation (2) for the steel part and Equation (3) for the concrete part.

(2) $$d_{i}^{S}-d_{i+1}^{S}=\frac{\sum_{j=1}^{i}F_{j}∙L_{i}}{E_{S}A_{S(i+1)}}$$

(3) $$d_{i}^{C}-d_{i+1}^{C}=\frac{(N-\sum_{j=1}^{i}F_{j})∙L_{i}}{E_{C}A_{C(i+1)}}$$

By associating the deformation coordination equation for the steel section of each segment within the entire SCJ according to Equation (3), a set of equations containing n equations can be obtained, as shown in Equation (4):

(4) $$\left\{\begin{array}{c} \begin{array}{c} d_{1}^{S}-d_{2}^{S}=\frac{F_{1}L_{1}}{E_{S}A_{S2}}\\ d_{2}^{S}-d_{3}^{S}=\frac{F_{1}+F_{2}}{E_{S}A_{S3}}L_{2}\\ \vdots \;\\ d_{i}^{S}-d_{i+1}^{S}=\frac{\sum_{j=1}^{i}F_{j}}{E_{S}A_{S(i+1)}}L_{i}\\ \vdots \;\\ d_{n}^{S}-d_{n+1}^{S}=\frac{\sum_{j=1}^{n}F_{j}}{E_{S}A_{S(n+1)}}L_{n} \end{array} \end{array}\right.$$

The deformation coordination equation for the concrete part of each section within the entire SCJ is associated according to Equation (3), which also yields a set of equations containing n equations, as shown in Equation (5):

(5) $$\left\{\begin{array}{c} \begin{array}{c} d_{1}^{C}-d_{2}^{C}=\frac{\left(N-F_{1}\right)}{E_{C}A_{C2}}L_{1}\\ d_{2}^{C}-d_{3}^{C}=\frac{(N-\sum_{j=1}^{2}F_{j})}{E_{C}A_{C3}}L_{2}\\ \vdots \;\\ d_{i}^{C}-d_{i+1}^{C}=\frac{(N-\sum_{j=1}^{i}F_{j})∙L_{i}}{E_{C}A_{C(i+1)}}\\ \vdots \;\\ d_{n}^{C}-d_{n+1}^{C}=\frac{(N-\sum_{j=1}^{n}F_{j})∙L_{n}}{E_{C}A_{C(n+1)}} \end{array} \end{array}\right.$$

The shear connection in paragraph *i* is equivalent to the equivalent spring of the steel structure and the concrete structure to transfer the axial force and the stiffness, from which the load deformation coordination equation of the equivalent spring can be obtained, as shown in Equations (6)–(8):

(6) $$F_{i}=K_{i}\Delta x_{i}$$

(7) $$\Delta x_{i}=\frac{\left(d_{i}^{C}+d_{i+1}^{C})+(d_{i}^{S}-d_{i+1}^{S}\right)}{2}$$

(8) $$K_{i}=K_{di}+K_{pi}$$

The stiffness of a single shear stud is calculated according to Equation (9) [34], where *d*_s_ is the diameter of the root of the shear stud.

(9) $$K_{d}=0.32d_{s}E_{S}^{0.25}E_{C}^{0.75}$$

The stiffness of a single PBL shear connection is calculated according to Equation (10) [21]. *f*_ck_ is the standard value of the compressive strength of the concrete, *d*_k_ is the diameter of the opening of the PBL shear connection, and *d*_p_ is the diameter of the penetrating reinforcement.

(10) $$K_{p}=23.4\sqrt{(d_{k}-d_{p})d_{p}E_{C}f_{ck}}$$

The load deformation relationship equation for the equivalent spring in each section of the entire lattice chamber can also be obtained from a set of equations containing n equations, as shown in Equation (11), by associating Equations (6) and (7):

(11) $$\left\{\begin{array}{c} F_{1}=0.5K_{1}\left[\left(d_{1}^{C}+d_{2}^{C})-(d_{1}^{S}+d_{2}^{S}\right)\right]\\ \vdots \\ F_{i}=0.5K_{i}\left[\left(d_{i}^{C}+d_{i+1}^{C})-(d_{i}^{S}+d_{i+1}^{S}\right)\right]\\ \vdots \\ F_{n}=0.5K_{n}\left[\left(d_{n}^{C}+d_{n+1}^{C})-(d_{n}^{S}+d_{n+1}^{S}\right)\right] \end{array}\right.$$

At the rear bearing plate, the concrete is only locally compressed at the edge of the bearing plate anchorage edge and the prestressing tendon anchor plate [35], and the concrete end as a whole still has axial displacement, so the longitudinal support stiffness of the bearing plate to the concrete is expressed, as shown in Equation (12):

(12) $$K_{hc}=E_{C}A_{zc}/t_{hc}$$

*A*_zc_ refers to the local bearing area of the concrete at the bearing plate. It is calculated as the projected area of the bearing plate on the steel box beam side of the support plate and anchor plate cross-section, projected onto the contact surface of the bearing plate and concrete at a 45° angle. This calculation subtracts the projected area of the steel member in that region. Additionally, *t*_hc_ represents the thickness of the rear bearing plate.

According to the deformation conditions at the rear bearing plate, Equations (13) and (14) can be obtained:

(13) $$d_{n+1}^{S}=0$$

(14) $$d_{n+1}^{C}=\frac{F_{hc}}{K_{hc}}=\frac{N-\sum_{j=1}^{n}F_{j}}{K_{hc}}$$

In the above assortment, the steel part of the absolute displacement of the end of the unknown number is *d*_1s_,*d*_2s_,…*d*_is_,…*d*_ns_, a total of n unknowns; the concrete part of the absolute displacement of the end of the unknown number is *d*_1c_,*d*_2c_,…*d*_ic_,…*d*_nc_, a total of n unknowns; each section of the shear key of the load is carried by the unknown number *F*_1_,*F*_2_,…*F*_i_,…*F*_n_, a total of n unknowns; and Formulas 4, 5, and 11 are for a system of equations, where the total number of unknowns is 3n and the total number of equations is 3n, which can be solved. All the unknowns, in addition to the parameters in this system of equations, shall be calculated by associating Equations (8)–(10) and Equations (12)–(14). The joint set of equations is essentially the deformation and load coordination equations of the SCJ based on the deformation coordination conditions of the equivalent spring, which is called the deformation coordination theoretical model, as each segment shear key is equivalent to an equal stiffness spring.

### 5.3. Solution of the Deformation Coordination Theory Model

The above deformation coordination theoretical model is derived from a large number of steps, which are converted into an equivalent form for ease of calculation and divided into the following three calculation steps:

1. Divide the SCJ into n sections, calculate the equivalent stiffness *K*_i_ of each section according to Equations (8)–(11), and obtain the end displacement *d*^s^_n+1_ of the steel and the stiffness *K*_hc_ of the rear bearing plate according to Equations (12) and (13);
2. Combining Equations (4), (5), and (11), the following derivations and simplifications are made:

Without loss of generality, for segment *i*, the following two equations are satisfied:

(15) $$\frac{E_{S}A_{S(i+1)}}{L_{i}}(d_{i}^{S}-d_{i+1}^{S})-K_{1}\Delta x_{1}-K_{2}\Delta x_{2}-\cdots \cdots -K_{i}\Delta x_{i}=0$$

(16) $$\frac{E_{C}A_{C(i+1)}}{L_{i}}(d_{i}^{C}-d_{i+1}^{C})+K_{1}\Delta x_{1}+K_{2}\Delta x_{2}+\cdots \cdots +K_{i}\Delta x_{i}=N$$

Similarly, Equation (14) can be changed to:

(17) $$K_{hc}d_{n+1}^{C}+K_{1}\Delta x_{1}+K_{2}\Delta x_{2}+\cdots \cdots +K_{i}\Delta x_{i}=N$$

Substitute Equation (7) with Equations (15)–(17) above, and make *i* = 1, 2, 3,……, n in Equations (15) and (16), respectively. The 2*n* equations can be obtained by combining Equations (13) and (17) to form a linear system of equations with 2*n* + 2 equations and 2*n* + 2 unknown quantities.

$$\left\{\begin{array}{l} K_{1}d_{1}^{C}+K_{1}d_{2}^{C}-(K_{1}+\frac{2E_{S}A_{S2}}{L_{1}})d_{1}^{S}-(K_{1}-\frac{2E_{S}A_{S2}}{L_{1}})d_{2}^{S}=0\\ K_{1}d_{1}^{C}+(K_{1}+K_{2})d_{2}^{C}+K_{2}d_{3}^{C}-K_{1}d_{1}^{S}-(K_{1}+K_{2}+\frac{2E_{S}A_{S3}}{L_{2}})d_{2}^{S}-(K_{2}-\frac{2E_{S}A_{S3}}{L_{2}})d_{3}^{S}=0\\ \begin{array}{ccc} & & \vdots \end{array}\\ K_{1}d_{1}^{C}+(K_{1}+K_{2})d_{2}^{C}+(K_{2}+K_{3})d_{3}^{C}+\cdots +(K_{i-1}+K_{i})d_{i}^{C}+K_{i}d_{i+1}^{C}-K_{1}d_{1}^{S}-(K_{1}+K_{2})d_{2}^{S}-\\ (K_{2}+K_{3})d_{3}^{S}-\cdots -(K_{i-2}+K_{i-1})d_{i-1}^{S}-(K_{i-1}+K_{i}+\frac{2E_{S}A_{Si+1}}{L_{i}})d_{i}^{S}-(K_{i}-\frac{2E_{S}A_{Si+1}}{L_{i}})d_{i+1}^{S}=0\\ \begin{array}{ccc} & & \vdots \end{array}\\ K_{1}d_{1}^{C}+(K_{1}+K_{2})d_{2}^{C}+(K_{2}+K_{3})d_{3}^{C}+\cdots +(K_{n-1}+K_{n})d_{n}^{C}+K_{n}d_{n+1}^{C}-K_{1}d_{1}^{S}-(K_{1}+K_{2})d_{2}^{S}-\\ (K_{2}+K_{3})d_{3}^{S}-\cdots -(K_{n-2}+K_{n-1})d_{n-1}^{S}-(K_{n-1}+K_{n}+\frac{2E_{S}A_{Sn+1}}{L_{n}})d_{n}^{S}-(K_{n}-\frac{2E_{S}A_{Sn+1}}{L_{n}})d_{n+1}^{S}=0 \end{array}\right.$$

$$\left\{\begin{array}{l} (K_{1}+\frac{2E_{C}A_{C2}}{L_{1}})d_{1}^{C}+(K_{1}-\frac{2E_{C}A_{C2}}{L_{1}})d_{2}^{C}-K_{1}d_{1}^{S}-K_{1}d_{2}^{S}=2N\\ K_{1}d_{1}^{C}+(K_{1}+K_{2}+\frac{2E_{C}A_{C3}}{L_{2}})d_{2}^{C}+(K_{2}-\frac{2E_{C}A_{C3}}{L_{2}})d_{3}^{C}-K_{1}d_{1}^{S}-(K_{1}+K_{2})d_{2}^{S}-K_{2}d_{3}^{S}=2N\\ \begin{array}{ccc} & & \vdots \end{array}\\ K_{1}d_{1}^{C}+(K_{1}+K_{2})d_{2}^{C}+(K_{2}+K_{3})d_{3}^{C}+\cdots +(K_{i-2}+K_{i-1})d_{i-1}^{C}+(K_{i-1}+K_{i}+\frac{2E_{C}A_{Ci+1}}{L_{i}})d_{i}^{C}+\\ (K_{i}-\frac{2E_{C}A_{Ci+1}}{L_{i}})d_{i+1}^{C}-K_{1}d_{1}^{S}-(K_{1}+K_{2})d_{2}^{S}-(K_{2}+K_{3})d_{3}^{S}-\cdots -(K_{i-1}+K_{i})d_{i}^{S}-K_{i}d_{i+1}^{S}=2N\\ \begin{array}{ccc} & & \vdots \end{array}\\ K_{1}d_{1}^{C}+(K_{1}+K_{2})d_{2}^{C}+(K_{2}+K_{3})d_{3}^{C}+\cdots +(K_{n-2}+K_{n-1})d_{n-1}^{C}+(K_{n-1}+K_{n}+\frac{2E_{C}A_{Cn+1}}{L_{n}})d_{n}^{C}+\\ (K_{n}-\frac{2E_{C}A_{Cn+1}}{L_{n}})d_{n+1}^{C}-K_{1}d_{1}^{S}-(K_{1}+K_{2})d_{2}^{S}-(K_{2}+K_{3})d_{3}^{S}-\cdots -(K_{n-1}+K_{n})d_{n}^{S}-K_{n}d_{n+1}^{S}=2N\\ \begin{array}{cc} & d_{n+1}^{S}=0 \end{array}\\ K_{1}d_{1}^{C}+(K_{1}+K_{2})d_{2}^{C}+(K_{2}+K_{3})d_{3}^{C}+\cdots +(K_{n-2}+K_{n-1})d_{n-1}^{C}+(K_{n-1}+K_{n}+)d_{n}^{C}+\\ (K_{n}+2K_{hc})d_{n+1}^{C}-K_{1}d_{1}^{S}-(K_{1}+K_{2})d_{2}^{S}-(K_{2}+K_{3})d_{3}^{S}-\cdots -(K_{n-1}+K_{n})d_{n}^{S}-K_{n}d_{n+1}^{S}=2N \end{array}\right.$$

3. Write the system of equations in step 2 in matrix form:
  $$\left[K\right]\left[D\right]=\left[F\right]$$
  [*K*] is the generalized stiffness matrix.
  $$\left[D\right]={\left[\begin{array}{cccccc} d_{1}^{C} & \cdots & d_{n+1}^{C} & d_{1}^{S} & \cdots & d_{n+1}^{S} \end{array}\right]}^{T}$$
  $$\left[F\right]={\left[\begin{array}{cccccccc} 0 & \cdots & 0 & 2N & \cdots & 2N & 0 & 2N \end{array}\right]}_{}^{T}$$

By solving the matrix, the displacements of the concrete structure and steel member at each section can be determined. These displacements can be substituted into Equation (11) to calculate the shear force in each section of the shear member. Afterward, the direct transfer of the axial force by the bearing plate, *F*_hc_, can be determined using Equation (14). Consequently, by performing an inverse analysis, the changes in axial forces along the longitudinal bridge direction for both the concrete and steel members can be obtained, along with the proportion of axial force transfer through each path.

### 5.4. Validation of the Calculation Method

To validate the accuracy of the deformation coordination theory model in calculating the transfer ratio of the SCJ structure, the model was applied to the calculation of the SCJ test model of the cross-Yujiang railway bridge in this study. The calculated results were then compared with the analysis results obtained from the ABAQUS finite element model.

**Figure 22:**
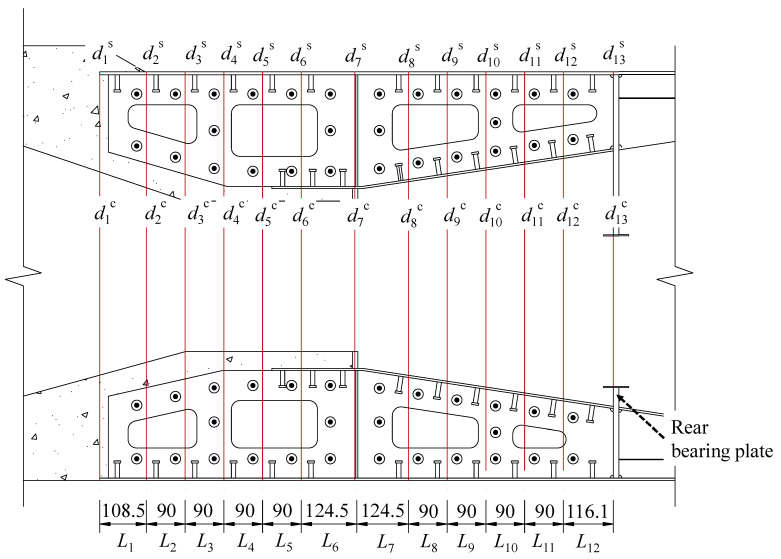
Simplified calculation diagram of the SCJ structure cross-Yujiang railway bridge (unit: mm).

Figure 22 illustrates the schematic diagram of the lattice chamber model used for the combined section of the cross-Yujiang railway bridge. The combined section is divided into 12 sections along the longitudinal direction. The model parameters for each section are presented in Table 11. The total load applied to the lattice chamber model is *N* = 2248 kN, and the equivalent support stiffness of the bearing plate is *K*_hc_ = 199284.18 kN/mm.

The parameter values in Table 11 were used to substitute the system of equations presented in this paper. This system is a linear system consisting of 26 equations and 26 unknowns. By solving this system of equations, the section displacements and shear forces of each section in the concrete structure and steel members were determined. The calculated results are provided below.

$$\begin{array}{ccc} \left\{\begin{array}{l} d_{1}^{C}=\begin{array}{l} 37.52\;\mu m \end{array}\\ d_{2}^{C}=34.43\;\mu m\\ d_{3}^{C}=32.04\;\mu m\\ d_{4}^{C}=29.75\;\mu m\\ d_{5}^{C}=27.56\;\mu m\\ d_{6}^{C}=25.44\;\mu m\\ d_{7}^{C}=22.61\;\mu m\\ d_{8}^{C}=19.04\;\mu m\\ d_{9}^{C}=16.39\;\mu m\\ d_{10}^{C}=13.69\;\mu m\\ d_{11}^{C}=10.98\;\mu m\\ d_{12}^{C}=8.34\;\mu m\\ d_{13}^{C}=5.44\;\mu m \end{array}\right. & \left\{\begin{array}{l} d_{1}^{S}=\begin{array}{l} 33.12\;\mu m \end{array}\\ d_{2}^{S}=31.95\;\mu m\\ d_{3}^{S}=30.52\;\mu m\\ d_{4}^{S}=28.69\;\mu m\\ d_{5}^{S}=26.58\;\mu m\\ d_{6}^{S}=24.71\;\mu m\\ d_{7}^{S}=21.83\;\mu m\\ d_{8}^{S}=18.48\;\mu m\\ d_{9}^{S}=15.87\;\mu m\\ d_{10}^{S}=13.05\;\mu m\\ d_{11}^{S}=9.89\;\mu m\\ d_{12}^{S}=6.25\;\mu m\\ d_{13}^{S}=0.00\;\mu m \end{array}\right. & \left\{\begin{array}{l} F_{1}=215.30\;\mathrm{kN}\\ F_{2}=114.73\;\mathrm{kN}\\ F_{3}=97.84\;\mathrm{kN}\\ F_{4}=76.11\;\mathrm{kN}\\ F_{5}=55.71\;\mathrm{kN}\\ F_{6}=63.64\;\mathrm{kN}\\ F_{7}=57.80\;\mathrm{kN}\\ F_{8}=37.38\;\mathrm{kN}\\ F_{9}=41.74\;\mathrm{kN}\\ F_{10}=73.86\;\mathrm{kN}\\ F_{11}=108.11\;\mathrm{kN}\\ F_{12}=275.91\;\mathrm{kN} \end{array}\right. \end{array}$$

**Figure 23:**
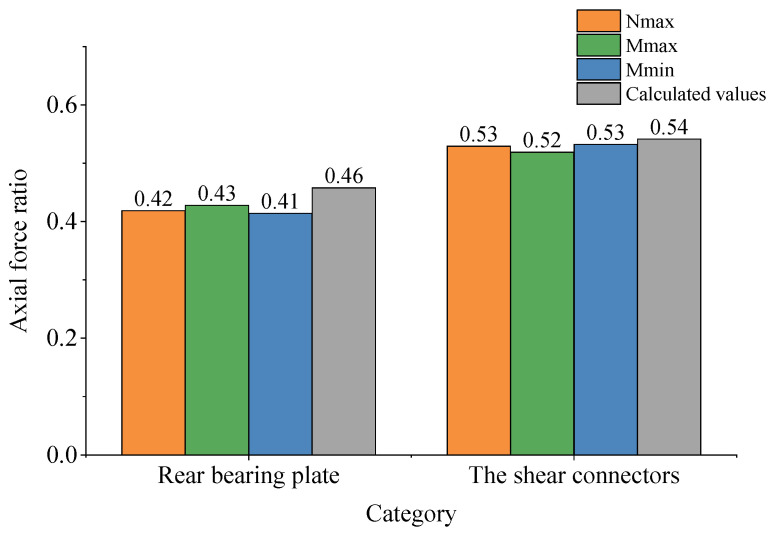
The ratio of axial force transfer for the rear bearing plate and shear connectors.

Based on the shear force analysis of each section, the specific ratio of force transfer between the bearing plate, shear connectors, concrete structure, and steel structure can be determined along the longitudinal direction of the bridge. A comparison between the finite element results and the simplified calculation results for the axial force transfer ratio of the rear bearing and shear connectors is illustrated in Figure 23.

Based on the analysis in Figure 23, it can be concluded that the axial force transfer ratio of the shear connectors shows good agreement between the finite element and simplified calculation results. However, the simplified calculation results for the rear bearing plate are slightly higher than the finite element results. This discrepancy is attributed to the omission of friction effects between the steel mix and the force transfer of the front bearing plate. Nevertheless, the conservative nature of the higher axial force transfer ratio for the rear bearing plate ensures the safety of its design. Therefore, this method can be effectively employed for estimating the axial force transfer ratio, calculating and designing the shear connectors, and conducting local verification of the bearing plate.

## 6. Conclusions

Based on the conducted investigations, the following conclusions can be drawn:

(1) The tested steel–concrete joint model, which did not consider the shear torsion effect and was subjected to 1.0 times the design load, exhibited continuous and smooth vertical deformation. The slip between the steel and concrete was minimal and linearly increased. Moreover, all members remained in an elastic working state even when subjected to 1.6 times the load. These findings indicate that the tested SCJ model possesses high safety, reliability, and an ample safety reserve.
(2) The measured data in the test model aligned well with the theoretically calculated values through finite element analysis, affirming the reliability of the finite element model for further analysis of the test.
(3) Through the finite element analysis of the test model, it was observed that the force transmission ratios of the steel–concrete joint members did not exhibit significant changes under different cases. They considered axial force and bending moment but excluded shear force and torsion. The rear bearing plate emerged as the primary axial force transfer member and was responsible for approximately 42% of the axial force transmission. The front bearing plate played a minor role, accounting for only about 7% of the transfer. The force transfer in other sections did not exhibit abrupt changes, indicating relatively smooth force transmission within the steel–cement combination zone and steel–cement transition zone.
(4) A simplified calculation model for the axial force transfer ratio of the steel–concrete joint was proposed based on the deformation coordination theory. The results of the simplified calculation demonstrated a close agreement between the axial force transfer ratio of the shear connectors obtained through finite element analysis and the simplified calculation. However, the simplified calculation yielded higher values for the rear bearing plate compared to the finite element results. Nevertheless, the higher transfer ratio for the rear bearing plate provides an added safety margin for its design. Therefore, the proposed method has valuable implications for estimating the steel–concrete joint transfer ratio, calculating and designing shear connectors, and conducting local verification of the bearing plate.

It is important to note that this study focuses solely on the effects of axial force and bending moment while excluding considerations for shear, torsion, dynamic loads, and other load types. Therefore, future research is required to investigate the mechanical behavior of steel–concrete joints under different loading conditions. Furthermore, due to limitations in the testing equipment, this study only examines the elastic stage and does not explore the failure modes and failure loads of steel–concrete joints. Future studies should enhance the loading methods and equipment to comprehensively investigate the failure modes and failure loads of steel–concrete joints. Additionally, this study primarily emphasizes the overall model testing of steel–concrete joint structures and does not specifically analyze the load-bearing behavior of internal shear connectors. Consequently, further research is necessary to investigate the specific mechanical behavior of internal shear connectors. Furthermore, the proposed simplified calculation method for force transfer ratios solely considers the force transfer mechanism of shear keys and backup plates, neglecting the impact of other structural components (such as front-end plates and friction between steel and concrete). Given the complex stress state of steel–concrete joints, future research is warranted to consider the force transfer mechanisms of other structural components and develop more precise calculation algorithms for force transfer ratios.

## Figures and Tables

**Table 1:** Top 10 main spans of hybrid girder cable-stayed bridges in the world.

Bridge Name	Country	Main Span (m)	Year
Russian Island Bridge	Russia	1104	2012
Stonecutters Bridge	China	1018	2009
Qingshan Yangtze River Bridge	China	938	2020
Erdong Yangtze River Bridge	China	926	2010
Jiayu Yangtze River Highway Bridge	China	920	2019
Dodoro Bridge	Japan	890	1999
Normandy Bridge	France	856	1995
Chizhou Yangtze River Highway Bridge	China	828	2019
Shishou Yangtze River Bridge	China	820	2019
Jiujiang Second Bridge	China	818	2013

**Table 2:** The similarity between the physical quantities of the test model and the real bridge.

Bridge Name	Parameter Similarity Ratio
Model size	1/5
Cross-sectional area	1/25
Cross-sectional moment of inertia	1/625
Axial force	1/25
Bending moment	1/125
Stress	1/1

**Table 3:** Comparison of test model steel plates and shear connectors with the actual bridge.

Component	Variable	Actual Bridge	Test Model
Top plate	Plate thickness	33 mm	6 mm
Base plate	Plate thickness	30 mm	6 mm
Center web	Plate thickness	30 mm	6 mm
Center side web	Plate thickness	30 mm	6 mm
Side webs	Plate thickness	30 mm	6 mm
Bearing plate	Plate thickness	64 mm	12 mm
PBL connectors	Diameter of the steel plate opening	60 mm	24 mm
PBL connectors	Diameter of penetration reinforcement	25 mm	10 mm
Shear studs	Specification	ϕ22 × 150 mm	ϕ10 × 40 mm

**Table 4:** Comparison of test model material and the actual bridge.

Material	Actual Bridge	Test Model
Steel	Q345qD	Q355B
Concrete	C60	C60
Steel bars	HPB300	HPB300
Steel bars	HRB400	HRB400
Shear studs	ML15	ML15

**Table 5:** Test results of test steel model material properties.

Steel Plate Thickness (mm)	Yield Strength (Mpa)	Tensile Strength (Mpa)	Elongation after Fracture (%)
4	463	568	30
6	433	479	28.5
8	409	498	30.5
12	434	570	27.5

**Table 6:** Test results of test model concrete material properties.

Serial Number	Length (mm)	Width (mm)	Height (mm)	Damage Load (kN)	Compressive Strength (Mpa)	Average Compressive Strength (Mpa)
1	148	147	148	1519.6	69.8	69.1
2	149	149	152	1468.4	66.1	
3	148	150	150	1580.5	71.2	

**Table 7:** Internal forces of the actual bridge control section under the action of different work conditions.

Type of Working Condition	Axial Force (kN)	Bending Moment (kN-m)	Shear Force (kN)	Torque (kN-m)
Maximum shaft force	−74,673.57	−4228.85	−22,489.47	−131.57
Minimum shaft force	−54,027.58	−657.18	40,272.85	−136.91
Maximum positive shear	−63,532.42	701.91	51,729.39	362.86
Maximum negative shear	−64,686.62	−6071.63	−22,770.58	−631.31
Maximum positive bending moment	−56,062.51	−571.15	74,856.05	362.86
Maximum negative bending moment	−72,461.53	−4366.84	−54,962.33	−131.32

**Table 8:** Total force applied by the jack under different load cases (unit: kN).

Load Case	The Total Force of the Top Jack	The Total Force of the Bottom Jack
1.0 times the maximum axial force (1.0 Nmax)	1255.38	1731.56
1.6 times the maximum axial force (1.6 Nmax)	2008.61	2770.50
1.0 times the maximum positive bending moment (1.6 Mmax)	1794.00	448.50
1.6 times the maximum positive bending moment (1.6 Mmax)	2870.40	717.60
1.0 times the maximum negative bending moment (1.6 Mmin)	899.52	1998.94
1.6 times the maximum negative bending moment (1.6 Mmin)	1439.24	3198.30

**Table 9:** Load conditions (unit: kN).

Load Case	Load Level	Single Top Jack Applied Load	Single Bottom Jack Applied Load
The maximum axial force	0.2 P	25.11	34.63
	0.4 P	50.22	69.26
	0.6 P	75.32	103.89
	0.8 P	100.43	138.52
	1.0 P	125.54	173.16
	1.2 P	150.65	207.79
	1.4 P	175.75	242.42
	1.6 P	200.86	277.05
The maximum positive bending moment	0.2 P	35.88	8.97
	0.4 P	71.76	17.94
	0.6 P	107.64	26.91
	0.8 P	143.52	35.88
	1.0 P	179.4	44.85
	1.2 P	215.28	53.82
	1.4 P	251.16	62.79
	1.6 P	287.04	71.76
The maximum negative bending moment	0.2 P	17.99	39.98
	0.4 P	35.98	79.96
	0.6 P	53.97	119.94
	0.8 P	71.96	159.92
	1.0 P	89.95	199.89
	1.2 P	107.94	239.87
	1.4 P	125.93	279.85
	1.6 P	143.92	319.83

**Table 10:** Axial force transfer ratio of steel and concrete structures.

Case	Type	Ⅴ-Right	Ⅴ-Left	Ⅳ	Ⅲ-Right	Ⅲ-Left	Ⅱ	Ⅰ
1.0 Nmax	Concrete	0	41.90%	55.49%	58.70%	63.85%	75.97%	100%
	Steel	100%	58.10%	44.51%	41.30%	36.15%	24.03%	0
1.0 Mmax	Concrete	0	42.81%	55.99%	58.89%	64.15%	76.82%	100%
	Steel	100%	57.19%	44.01%	41.11%	35.85%	23.18%	0
1.0 Mmin	Concrete	0	41.43%	55.02%	57.68%	63.01%	75.04%	100%
	Steel	100%	58.57%	44.98%	42.32%	36.99%	24.96%	0

**Table 11:** Calculation results of the main parameters of each section.

Segment i	Length *L_i_* (mm)	Number of Shear Studs	Number of PBL	Total Stiffness (kN/mm)	*A*_c*i*+1_ (mm^2^)	*A*_s*i*+1_ (mm^2^)
1	108.5	153	54	62,595.41	1,979,677.02	95,149.77
2	90	124	54	57,403.47	2,006,623.15	98,698.38
3	90	128	81	75,721.33	1,989,173.64	100,143.78
4	90	219	54	74,411.55	1,989,173.73	102,380.3
5	90	167	54	65,101.87	1,989,173.73	128,281.52
6	124.5	174	81	83,956.82	1,989,173.73	128,268.09
7	124.5	184	81	85,747.14	1,515,175.06	120,476.9
8	90	188	54	68,861.55	1,445,307.86	118,083.41
9	90	205	54	71,905.10	1,375,440.66	115,656.62
10	90	184	81	85,747.14	1,305,573.46	113,273.01
11	90	184	54	68,145.42	1,235,706.26	110,836.35
12	116.1	213	54	73,337.36	1,144,551.06	107,727.54

## Data Availability

Not applicable.
